# Tumor Growth Rate in Neuroendocrine Neoplasms: An Additional Tool for Treatment Strategies?

**DOI:** 10.3390/medicina61101852

**Published:** 2025-10-16

**Authors:** Roberta Modica, Alessia Liccardi, Elio Benevento, Roberto Minotta, Gianfranco Di Iasi, Massimo Di Nola, Michele Coletta, Annamaria Colao

**Affiliations:** Endocrinology, Diabetology and Andrology Unit, Department of Clinical Medicine and Surgery, Federico II University of Naples, 80131 Naples, Italy; alessia.liccardi@yahoo.com (A.L.); elio.benevento@gmail.com (E.B.); robertominotta@gmail.com (R.M.); gianfrancodiiasi@gmail.com (G.D.I.); massimodinola96@gmail.com (M.D.N.); michele99coletta@gmail.com (M.C.); colao@unina.it (A.C.)

**Keywords:** neuroendocrine neoplasms, neuroendocrine tumors, tumor growth rate, somatostatin analog, target therapy, radioligand therapy, chemotherapy

## Abstract

*Background and Objectives*: Neuroendocrine neoplasms (NENs) are rare, mainly gastro-entero-pancreatic tumors with heterogeneous biology and multiple therapeutic options. Assessing treatment response remains challenging. Standard evaluation relies on RECIST 1.1, although its limitations are well recognized. Tumor growth rate (TGR), defined as the monthly percentage change in tumor size between two imaging assessments, has been proposed as a dynamic parameter to complement conventional criteria. This review explores the role of TGR in NEN. *Results*: Two different evaluations of TGR, once conducted between baseline diagnostic scan and a radiological assessment 12–24 weeks after (TGR0), and another conducted between baseline scan and a diagnostic evaluation three months after (TGR3m), proved to be well correlated to progression free survival (PFS) in G1 and low-G2 NEN, with cut off of 4%/month and 0.8%/month, respectively. *Conclusions*: TGR offers additional insights into tumor kinetics and may help refine treatment monitoring in NEN. While retrospective evidence supports its prognostic utility, prospective studies are required to validate TGR as a standard clinical tool.

## 1. Introduction

Neuroendocrine neoplasms (NEN) are rare tumors arising from neuroendocrine tissue, with a ubiquitous distribution and an increasing incidence up to 6.98/100,000 patients, with the highest annual percentage increase for NEN originating in the gastro-entero-pancreatic tract (GEP-NEN; 3.56/100,000) and the lungs (L-NEN; 1.49/100,000) [1,2]. Most GEP-NEN are well-differentiated tumors (NET) and slow-growing, classified by the World Health Organization (WHO) into G1, G2, and G3 based on the mitotic index and Ki67 (G1: Ki67 < 3% and number of mitoses < 2/10 high-power fields; G2: Ki67 between 3% and 20% and number of mitoses between 2 and 20/10 high-power fields; G3: Ki67 > 20% and number of mitoses > 20/10 high-power fields) [3]. Noteworthy, the indolent nature of the disease often leads to diagnostic delay, with 40–50% of patients presenting with metastases at diagnosis, thus necessitating systemic medical therapy [4,5].

Therapeutic choice mainly depends on tumor site, differentiation, disease extension, and patient’s clinical condition [6]. Currently, therapeutic response is assessed using RECIST 1.1 criteria or, less commonly, through Choi criteria: they both rely on the comparison of high-resolution imaging studies, such as computed tomography (CT) or magnetic resonance imaging (MRI), performed at baseline and at the most recent follow-up of the same modality [7,8,9]. According to RECIST 1.1, a maximum of five target lesions can be selected for evaluation, with no more than two lesions per organ. Lesions are measured in their longest diameter, except for lymph nodes, where the shortest axis must be considered [7]. To qualify as target lesions, they must measure at least 10 mm in the longest diameter or 15 mm in the short axis for lymph nodes. A complete response (CR) is defined in both systems as the disappearance of all target lesions without evidence of new disease [7]. For partial response (PR), RECIST 1.1 requires at least a 30% reduction in the sum of diameters of target lesions [7]. In contrast, the Choi criteria define PR as either a reduction of at least 10% in tumor size or a decrease of at least 15% in tumor density, as measured in Hounsfield units on CT, provided that no new lesions appear and non-measurable disease does not progress [8,9]. Stable disease (SD) is defined similarly in both systems, indicating that the observed changes in tumor burden are insufficient to qualify either for partial response or for progression [8,9]. Finally, progressive disease (PD) under RECIST 1.1 corresponds to at least a 20% increase in the sum of diameters of target lesions or the appearance of new lesions [7]. According to the Choi criteria, progression is identified when there is at least a 10% increase in tumor size, combined with the absence of a density reduction sufficient for partial response, or when new lesions are detected [8,9]. The application of these criteria to NEN is limited by the typically indolent nature of most of these tumors; as a result, non-responders may be misclassified as having stable disease, emphasizing the need for earlier and more sensitive markers of therapeutic response [6,8,9]. Furthermore, the selection of target lesions is guided by a dimensional criterion (≥1 cm), but there are no guidelines for selecting two representative lesions per organ, which is crucial for the follow-up [10]. Additionally, RECIST criteria only consider the dimensional growth of target lesions, neglecting vascularization or the effects of locoregional therapies (including thermoablation/chemoembolization) or anti-angiogenic drugs (including sunitinib and cabozantinib) on the lesions, which might be underestimated or even unrecognized due to the presence of edema or necrosis [10,11]. To date, no standardized radiological tools are available to capture these aspects, highlighting the need to move beyond the limitations of RECIST criteria in therapeutic decision-making.

## 2. Tumor Growth Rate: The State of the Art

### 2.1. Definition

The concept of exponential growth of tumor resulting from the rate of cell division and the constant volume doubling time was first introduced by Collins et al. in 1956 [12]. The authors established that the rapid growth of the tumor was a hallmark to identify malignant lesions: indeed, a correlation between the tumor growth and survival was identified in a population of patients with breast cancer. In a multicentric Japanese study, three groups were formed based on growth rate, and the 5-year survival rate appeared to be significantly worse in the “rapid growing” group than “intermediate/low growing” groups [13]. Thus, the history of tumor growth rate (TGR) dates back to the 90s, even if only recently this morphological parameter has been used in NET field. The TGR has been proposed as a quantitative measurement based on two radiological assessments of the same modality that reflect the percentage dimensional variation per month [14]. TGR is obtained from the following mathematical formula:

TGR = 100 (e^(TG)^ − 1),

where TG = [3 × log(D2/D1)]/t; D2: tumor dimension obtained as the sum of the largest diameters of the target lesions according to RECIST 1.1 evaluated at time 2; D1: tumor dimension evaluated at time 1; t (time in months) = (date 2 *−* date 1 + 1)/30.44 [14,15].

### 2.2. Treatment Strategies in Neuroendocrine Neoplasms and Challenges in Response Evaluation

An accurate and early tool for treatment response assessment is essential, particularly in the evolving therapeutic landscape of NEN, where several new systemic agents are already available or currently under investigation and the optimal therapeutic sequence remains a matter of debate [6]. In well-differentiated NET (G1-G2) with evidence of active, unresectable disease, whether functioning or non-functioning, various guidelines agree on initiating long-acting somatostatin analogs (SSA) as first-line therapy (Table 1) [16,17], SSA were initially used for their antisecretory role in treating functioning NEN, then in PROMID and CLARINET studies they demonstrated an antiproliferative effect in GEP-NET of octreotide and lanreotide, respectively, significantly increasing progression-free survival (PFS) compared to placebo [16,18]. The efficacy of SSA is also demonstrated in treating NET arising in other organs, such as the lungs, and in managing patients with duodenopancreatic NET in the context of multiple endocrine neoplasia type 1 (MEN 1) syndrome, where early initiation of SSA can delay the timing of surgery [19]. However, SSAs rarely induce a complete or partial response according to RECIST 1.1 criteria but more frequently exert a cytostatic effect, leading to disease stabilization, thus requiring careful radiological evaluation [17].

Like SSA, radioligand therapy, also known as peptide receptor radionuclide therapy (RLT or PRRT), exploits the expression of somatostatin receptors on NET cells, using radiolabeled peptides to deliver a targeted cytotoxic effect [20]. The first prospective randomized trial showing the efficacy of RLT was NETTER 1, which enrolled 229 patients with advanced midgut NET. Patients receiving RLT in combination with standard-dose SSA achieved significantly longer PFS and improved symptom control, compared with patients treated with high-dose SSA [21]. Morphological response was evaluated exclusively by RECIST 1.1 criteria, essential for defining PFS and obtaining the overall response rate (ORR-18% in RLT Cohort vs. 3% in high-dose SSA cohort; *p* < 0.0004) [21]. Based on these findings, RLT is currently proposed as a second-line therapy for metastatic midgut G1-G2 NET and is recommended for early use upon progression from SSA in other GEP-NET [22,23]. More recently, the NETTER-2 trial demonstrated that first-line RLT combined with octreotide LAR significantly prolonged median PFS in patients with advanced G2-G3 GEP-NET, suggesting that RLT may represent a new standard of care in this setting [24]. In both NETTER-1 and NETTER-2, treatment efficacy was assessed exclusively by RECIST 1.1 criteria (Table 1). Radiological assessment is of particular importance during targeted therapy in NET, as treatment effects may include not only tumor shrinkage, but also changes such as necrosis and alterations in tumor density. Among the targeted agents currently used in clinical practice, everolimus, an inhibitor of the mammalian target of rapamycin (mTOR), and sunitinib, a tyrosine kinase inhibitor (TKI), have demonstrated significant efficacy and remain widely adopted [6]. The efficacy of everolimus, evaluated in terms of PFS and ORR versus placebo, has been demonstrated in the four RADIANT studies, leading to its approval for GEP-NET and lung NET progressing after first-line therapy (Table 1) [25,26]. The efficacy of sunitinib has been established in pancreatic NET (panNET) in placebo-controlled studies, in which treated patients achieved improved PFS (11.4 vs. 5.5 months) and overall survival (OS, 38.6 vs. 29.1 months) [27]. In a retrospective analysis of 10 patients previously included in the sunitinib phase III trial, only two patients were classified as responders after 4 weeks of treatment according to RECIST, whereas six achieved a partial response by Choi criteria and had an improved time to tumor progression compared with non-responders (Table 1) [28]. Choi criteria, which incorporate both tumor size and density on CT, were further assessed in 107 panNET patients treated with sunitinib, showing superior sensitivity to treatment effects compared with RECIST. Indeed, patients who achieved partial response increased from 12.8% (RECIST) to 47.4% (Choi). Moreover, 24% of patients defined as having progressive disease by Choi had stable disease with RECIST, underscoring the added value of density-based evaluation [29]. In the context of systemic therapies, the role of TGR is particularly intriguing, also with regard to chemotherapy. Cytotoxic agents are generally reserved for high-grade or rapidly progressive disease, where volumetric changes can be more evident than in indolent forms [30]. In this setting, TGR might provide additional information on tumor dynamics, complementing conventional radiological criteria. TGR may also be of interest in the context of locoregional therapies, including: transarterial embolization, chemoembolization, radioembolization, or ablative approaches, that often induce heterogeneous radiological changes, including necrosis, cavitation, and density modifications, that are not always adequately captured by RECIST criteria [31]. In these settings, TGR could provide a quantitative measure of the overall tumor kinetics before and after treatment, potentially distinguishing patients with biologically indolent disease from those with more aggressive tumor behavior.

Overall, while the therapeutic landscape of NEN is rapidly evolving, the optimal tools to assess treatment response are still debated. TGR represents a promising parameter that could complement conventional radiological criteria, but its applicability in NEN remains to be clarified. The following section will address the limited but growing body of studies exploring TGR specifically in this setting.

### 2.3. Tumor Growth Rate in Neuroendocrine Neoplasms: Evidence and Application

#### 2.3.1. Post Hoc Analysis of the CLARINET

The role of TGR in evaluating treatment response in NEN was first explored in a Post Hoc analysis of the CLARINET, a multicenter, phase III, double-blind, randomized study (204 patients: 101 intervention arm, 103 placebo) (Table 2) [32]. Patients in the intervention arm received lanreotide 120 mg subcutaneously every 4 weeks for up to 96 weeks or until disease progression (PD, evaluated through RECIST 1.0). A distinctive feature of this analysis was the inclusion of a radiological assessment 12–24 weeks after the baseline diagnostic scan, enabling calculation of baseline TGR (TGR0) before randomization. Patients with TGR0 ≤ 4%/month, irrespective of treatment allocation, achieved longer PFS than those with TGR0 > 4%/month. Notably, even among patients with TGR0 > 4%/month, lanreotide prolonged PFS compared with placebo (96.3 vs. 37.7 weeks). Univariate analysis identified the following items as significant prognostic factors for PFS: baseline disease status (progressive vs. stable), former NET treatment, Ki-67 index, and TGR0. Scatter plot analysis further showed that TGR generally decreased after lanreotide initiation, particularly in patients with evidence of PD at baseline: thus, among patients with PD at baseline (*n* = 11), 10 (90.9%) exhibited a reduction in TGR, while TGR remained largely stable in patients with baseline SD. Despite being retrospective, this analysis supports TGR as both a prognostic marker identifying patients at higher risk of early progression and a dynamic marker of treatment activity, offering a more refined evaluation of drug effect rather than the RECIST criteria. Indeed, patients classified as having SD by RECIST were often experiencing active tumor growth, with TGR0 values around 2–3% per month. Importantly, a threshold of 4%/month clearly separated patients into distinct prognostic groups, with those above this cutoff showing a significantly higher risk of early progression.

#### 2.3.2. The GREPONET Studies

**Figure 1:**
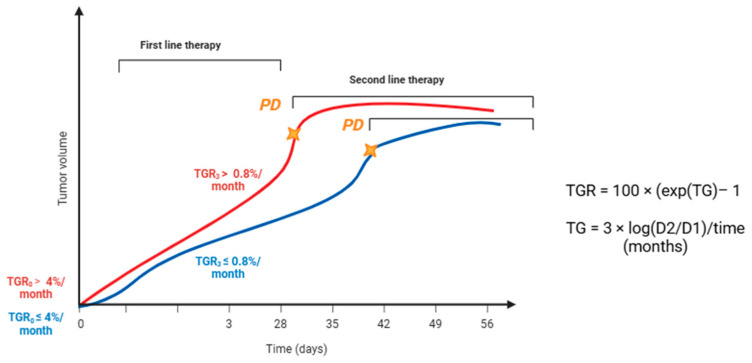
Evaluation of TGR0 and TGR3m. For G1 and G2 NET with a low proliferative index, TGR cut-offs at both time 0 and 3 months correlate with PFS and radiological response to treatment according to Lamarca et al. and Dromain et al. [15,32]. TGR was expressed as the percentage change in tumor volume over 1 month (%/month), TG = tumor growth, D1 = tumor size at date 1, D2 = tumor size at date 2, and time (months) = (date 2 − date 1 + 1)/30.44. Tumor size was determined using the sum of the longest diameters of target lesions only. Yellow stars stand for the occurrence of progression disease according to traditional evaluation trough RECTST 1.1 criteria.

The GREPONET study provided the most robust evidence to date that TGR can serve as an early radiological biomarker in patients with advanced GEP NET (Table 2) [15]. By retrospectively analyzing 222 patients with G1 or G2 small intestinal NET or panNET in advanced stage (not eligible for curative resection) across 7 international centers, the investigators demonstrated that TGR evaluated at three months (TGR3m) is a strongly predictive item of PFS. TGR0 did not differ significantly across tumor sites, grades, or treatment groups. By contrast, TGR3m outperformed RECIST3m in predicting 12-month progression, with greater reliability and less variability. A cut-off of 0.8%/month was identified as the most informative threshold: patients with TGR3m ≥ 0.8%/month had a significantly higher risk of early progression and shorter PFS compared with those below this threshold. Importantly, this finding remained consistent across multiple sensitivity analyses and was independent of the type of treatment received [15]. The prognostic value of TGR3m was further confirmed in the subgroup of patients who achieved only stable disease by RECIST criteria. Even in this context, TGR3m discriminated between patients with favorable versus poor outcomes, highlighting that TGR provides information that conventional RECIST fails to capture. TGR was also shown to be more accurate and less variable than RECIST-defined changes at 3 months, emphasizing its superiority as a dynamic measure of tumor kinetics. Pre-treatment TGR (TGR0) also carried prognostic significance: patients with TGR0 ≥ 4%/month experienced significantly shorter PFS compared with those with TGR0 < 4%/month, corroborating earlier findings from the CLARINET. Together, these results establish both TGR0 and TGR3m as meaningful indicators of tumor biology and clinical outcome. From a practical perspective, the study suggests that TGR could be used to tailor follow-up in clinical practice: patients with high TGR3m may require closer imaging surveillance, while those with low TGR3m could be monitored less frequently, thereby avoiding unnecessary radiation exposure and reducing patient burden (Figure 1). Even in the GREPONET study, there is evidence that TGR is a feasible, reproducible, and clinically informative biomarker in NET, serving both as a prognostic factor and a tool for refining radiological assessment beyond RECIST. Nevertheless, this study was retrospective and heterogeneous in terms of treatment exposures, and subgroup analyses were limited by small sample sizes [15].

In the subsequent retrospective GREPONET-2 study, the same research group pursued two main objectives: first, to evaluate whether standard treatments were associated with measurable changes in TGR (ΔTGR 3m-BL: TGR3m–TGR0), and second, to validate the prognostic value of TGR3m ≥ 0.8%/month on an independent cohort of patients with NET (Table 2) [33]. The analysis showed that the most pronounced reductions in ΔTGR 3m-BL occurred in patients treated with SSA, targeted therapies, and chemotherapy, although no statistically significant difference was observed among treatment classes. Conversely, a watch-and-wait approach did not influence TGR. In both univariate and multivariate analyses, only pancreatic primary tumors and high baseline TGR0 (≥4%/month) were independently associated with greater ΔTGR 3m-BL reduction, irrespective of tumor grade or treatment received. At the univariate level, ΔTGR 3m-BL correlated with PFS, but this association was lost in multivariate analysis. By contrast, a high TGR3m was consistently associated with shorter PFS, both in the original cohort and in the independent validation cohort, confirming the robustness of the 0.8%/month threshold as a prognostic factor [33]. Importantly, broader variability in TGR3m was observed in patients treated with chemotherapy or targeted agents, whereas no significant changes were detected in patients undergoing RLT. This lack of significance was likely related to the observation window, as RLT can induce delayed tumor shrinkage in the months following completion of therapy cycles. Taken together, GREPONET-2 consolidates the role of TGR3m as a dynamic and reproducible biomarker in NET, confirming its prognostic value across different patient populations (Figure 1). While ΔTGR 3m-BL may capture some treatment-related effects, it is TGR3m itself that most reliably correlates with long-term outcomes. These findings reinforce the superiority of TGR over RECIST for assessing tumor kinetics and support its incorporation into prospective studies and treatment evaluation frameworks.

#### 2.3.3. TGR in PRRT and Novel Approaches

Pettersson et al. (2021) investigated the value of TGR as a dynamic marker of treatment response in patients with panNET undergoing RLT with 177Lu-DOTATATE (Table 2) [34]. Sixty-seven patients were retrospectively analyzed, with TGR calculated at baseline, approximately 4.5 months, and 10 months after therapy beginning. The study demonstrated that the mean TGR before treatment was around 6% per month, reflecting active tumor progression, but decreased significantly to negative values after PRRT, indicating tumor shrinkage. Importantly, while baseline TGR and early changes at 4.5 months were not clearly predictive, patients who maintained a positive TGR at 10 months (≥0.5% per month) had markedly shorter PFS compared with those whose TGR was negative. OS was not significantly modified, but a low Ki-67 index (<10%) was also associated with PFS [34]. These findings suggest that RLT has a profound impact on tumor kinetics, with TGR capturing early reductions in growth that are not always evident with RECIST. Moreover, persistence of tumor growth during treatment identifies patients at risk of early progression, supporting the role of TGR as a complementary biomarker to conventional imaging criteria [35]. An alternative approach to evaluate TGR involves approximating tumor volume as an elliptical cylinder by measuring the major and minor axes of the elliptical base in the axial plane and the corresponding height in the coronal view. This geometric model may more faithfully represent the irregular shapes of GEP-NET lesions and requires only minimal additional radiologist time, without affecting acquisition protocol or patient burden. In a proof-of-concept study of 58 GEP-NET patients undergoing PRRT, cylindrical TGR (cTGR) was compared with conventional, sphere-based TGR. The cTGR demonstrated superior predictive performance for early disease progression: the ROC area under the curve (AUC) was 1.00 for cTGR versus 0.92 for TGR, and multivariate analysis confirmed a higher predictive coefficient (1.56 vs. 1.45, respectively) (Table 2) [36].

#### 2.3.4. Additional Retrospective Evidence

Wang et al. (2023) evaluated retrospectively the prognostic role of baseline TGR (TGR0) in 48 patients with advanced, well-differentiated G1–3 GEP-NET. Median baseline TGR was 4.8% per month, but values ranged widely, reflecting the marked heterogeneity of NET biology. TGR0 strongly correlated with pretreatment Ki-67 across the whole cohort and within the G3 subgroup, but not in G1-2 disease. Patients with high TGR0 (>11.7%/month) experienced a significantly shorter time to first therapy and reduced OS compared to those with low TGR0. Importantly, TGR0, but not grade defined by Ki-67 thresholds, predicted subsequent increases in Ki-67 on serial biopsies. Indeed, all patients with high TGR0 who underwent repeat biopsy showed Ki-67 escalation, whereas only half of those with low TGR0 did. This suggests that baseline TGR may non-invasively identify patients with occult biological aggressiveness or forthcoming grade progression (Table 2) [35]. The study also noted that high TGR0 was most common in panNET, while low TGR0 was more frequently observed in small bowel tumors, underlying differences in tumor behavior by primary site. Limitations include the small, single-institution cohort and retrospective design, as well as variability in imaging intervals. Nevertheless, the findings reinforce the value of TGR0 as a dynamic biomarker distinct from conventional grade, with potential to refine risk stratification, guide surveillance intensity, and prompt earlier repeat biopsy in patients with unexpectedly high growth rates [35]. Moreover, in a multicenter retrospective cohort study, Baechle et al. (2020) included 288 panNET with 2 pre-operative imaging executed at least 1 month apart: a surrogate evaluation of tumor growth rate, the standard growth rate (SGR, % growth/day) was calculated as reported below, where Di is the initial imaging, Df the final preoperative imaging, T-T0 the time between initial and final preoperative imaging studies (Table 2) [37].

SGR = 3 × ln (Di:Df)/(T − T0)

Interestingly, patients with a level of SGR over 90th percentile (0.93%/day) had a significantly shorter OS (*p* = 0.013) and disease-specific survival (*p* = 0.025) than people with SGR below 90th percentile.

These findings suggest that TGR could be considered a dynamic marker in NEN, providing prognostic information and capturing treatment effects earlier and more sensitively than RECIST. High baseline TGR may identify patients at risk of early progression, while early reductions in TGR under therapy reflect treatment benefit, irrespective of conventional radiological stability. Although evidence is still retrospective and heterogeneous, TGR appears reproducible across different cohorts and treatment settings, supporting its validation in prospective studies and its potential integration into clinical practice.

## 3. Discussion

TGR combines the RECIST sums of target lesions and the time between the tumor evaluations. RECIST are demanding criteria since an objective response requires at least a 30% reduction in tumor size, while any new lesion automatically results in progressive disease [10]. This may limit the suitability of these criteria for evaluating low-grade NET, since some responders and non-responders are both categorized as stable disease. The favorable effect of the treatment may therefore be underestimated, and conversely, the detection of tumor growth may be delayed. Nevertheless, TGR itself has some limitations. A key challenge is determining the optimal timing of radiological reassessment. The GREPONET-2 study applied strict 3-month follow-up intervals [33], whereas most guidelines recommend 6-month imaging in well-differentiated NET patients receiving first-line SSA. A 3-month interval may not be sufficient to provide reliable information, due to the indolent behavior of NETs and to the cytostatic effect of SSAs.

The GREPONET-2 study was affected by a selection bias: indeed, out of the 127 enrolled patients, 80 were G2 (62.99%), only 39 were G1 (30.71%), and only 7 were locally advanced (5.5%), while the remaining 120 were metastatic (94.49%). Additionally, the number of patients on SSA treatment (48/127, 37.8%) was almost equal to those on chemotherapy (41/127; 32.28%), differing from real-world cohorts [6,33]. Furthermore, neither CLARINET, GREPONET, nor GREPONET-2 enrolled G3 NET patients, making the applicability of TGR to this population uncertain. CLARINET and GREPONET-2 enrolled G2 NET but with Ki67 < 10%, while GREPONET included G2 NET patients with Ki67 between 3 and 20%, with a median of 8% [15,18,32]. Thus, proposed TGR cut-offs (TGR0, TGR3m) are mainly relevant to G1 and low-G2 NET, which tend to be less aggressive. Lastly, calculating TGR based on D1 and D2, as specified in the formula, presents the same challenges as RECIST 1.1 criteria regarding the choice of target lesions representative of the disease and standardization of measurements. Finally, calculating TGR based on linear measurements (D1, D2) inherits the same limitations as RECIST 1.1, such as the choice of target lesions and the reproducibility of measurements. A recent exploratory study proposed an integrated approach combining tumor growth and regression parameters, which appeared to improve correlation with PFS and facilitate radiological evaluation. However, this approach is not yet validated for routine use [38]. Considering that TGR quantified through tumor volume doubling time (DT) may suffer from several methodological limitations, including skewed distribution, dependence on measurement intervals and volume uncertainties, and the lack of definition when sequential measurements are equal a quantitative measure of growth rate, the specific growth rate (SGR, %/day), has been proposed as a more robust and statistically suitable parameter [37]. However, its applicability to NEN, particularly those with very slow growth, may be limited. In indolent tumors, small variations in volume measurement or imaging uncertainties can disproportionately affect SGR estimates, potentially leading to misleading conclusions. Regarding the use of TGR, no data are currently available on its relationship with the site of the primary tumor and OS. These aspects remain unmet needs in the treatment and monitoring of NET patients and require dedicated long-term studies to allow a specific assessment of survival outcomes and primary tumor characteristics. Indeed, the application of TGR has so far been restricted almost exclusively to panNEN and a few other GEP sites, without exploring its potential relevance in NENs of different origin, such as pulmonary or thymic.

TGR is proposed as an innovative tool in the constantly evolving diagnostic landscape of NEN, aiming to improve therapeutic management, with particular attention to quality of life [5,39]. In this context, its integration with translational biochemical evaluations, such as those involving miRNA, could represent a step forward in the development of a personalized diagnostic and care pathway [5,40].

## 4. Conclusions

TGR has emerged as a promising, yet still exploratory, tool in NEN treatment, offering insights into tumor kinetics that go beyond conventional radiological criteria. For G1 and low-G2 NET, it has been possible to define TGR cut-offs at both baseline and 3 months that correlate with PFS and radiological response. Patients with TGR0 (assessed 12–24 weeks from diagnosis without treatment) > 4%/month show shorter PFS following first-line therapy, particularly with SSA, and therefore may benefit from closer surveillance. For patients receiving targeted therapies or chemotherapy, calculation of TGR3m can identify therapeutic failure earlier than RECIST 1.1 when using a threshold of 0.8%/month (Figure 1). Conversely, response to RLT does not appear to be adequately captured by either TGR3m or TGR10. Across published studies, TGR has not correlated with Ki-67 and cannot replace it. Nonetheless, a TGR0 > 11.7%/month has been linked to an increased risk of Ki-67 escalation in metastatic sites compared to the primary tumor. While TGR during treatment may anticipate progression, potentially supporting treatment switches, it requires frequent radiological assessments, raising issues of feasibility and patient burden. The absence of prospective validation further underscores the need for caution. Early distinction between responders and non-responders would be of utmost importance to improve tailored management of patients, and the design of clinical trials, providing tools to define progression and PFS. TGR incorporation into prospective clinical trials and routine practice could potentially improve patient risk stratification, treatment evaluation, and surveillance strategies in advanced NET.

## Figures and Tables

**Table 1:** Evidence on the efficacy of current therapies in neuroendocrine neoplasms and response assessment challenges.

Therapy	Evidence/Key Trials	Main Findings	Response Assessment Challenges
Somatostatin analogs (SSA) (Octreotide, Lanreotide)	PROMID, CLARINET	Significant PFS benefit vs. placebo; efficacy also in lung NET and MEN1-related duodenopancreatic NET	Rare CR/PR by RECIST 1.1; mainly cytostatic effect (disease stabilization); careful radiological monitoring required
Radioligand therapy (PRRT/RLT) (^177Lu-DOTATATE)	NETTER-1, NETTER-2	Prolonged PFS and improved symptom control; 18% ORR vs. 3% with high-dose SSA; efficacy in both midgut and G2-G3 GEP-NET	Efficacy assessed only by RECIST 1.1; density or growth-rate changes not captured; TGR could provide earlier response detection
Targeted therapies (Everolimus, Sunitinib)	RADIANT trials (everolimus); Phase III sunitinib (panNET)	Everolimus: improved PFS in GEP and lung NET; Sunitinib: improved PFS (11.4 vs. 5.5 mo) and OS (38.6 vs. 29.1 mo)	RECIST may underestimate benefit; Choi criteria are more sensitive for sunitinib (PR 47.4% vs. 12.8% by RECIST); necrosis/density changes are not reflected in RECIST
Chemotherapy	Limited studies have been used in high-grade/progressive NET	More effective in aggressive disease; volumetric changes are more evident	Role of TGR exploratory; rapid TGR decrease may predict response, persistent TGR may indicate resistance
Locoregional therapies (embolization, chemoembolization, radioembolization, ablation)	Observational studies	Can induce necrosis, cavitation, density changes	RECIST is often inadequate; TGR may provide quantitative kinetics, but validation is limited due to multifocal disease and technical issues

PFS, progression-free survival; OS, overall survival; CR/PR, complete response/partial response; RECIST, Response Evaluation Criteria in Solid Tumors; TGR, tumor growth rate.

**Table 2:** Summary of studies on tumor growth rate in neuroendocrine tumors.

References	Study Type	Population	Main Findings on TGR	Reported TGR Thresholds/Values
[32]	Post hoc analysis of a randomized trial (CLARINET)	204 NET	TGR0 ≤ 4%/month, irrespective of treatment allocation, achieved longer PFS than those with TGR0 > 4%/month.	Even among patients with TGR0 > 4%/month, lanreotide prolonged PFS compared with placebo (96.3 vs. 37.7 weeks).
[33]	Proof-of-concept study	58 GEP-NET	Cylindrical TGR (cTGR) outperformed conventional TGR in predicting progression (ROC AUC 1.00 vs. 0.92).	No absolute cut-off reported; cTGR showed higher predictive accuracy.
[34]	Retrospective study	151 PanNET	TGR markedly decreased during PRRT; high pre-treatment TGR identified non-responders and correlated with shorter PFS.	Pre-PRRT TGR median ~ +2.3%/mo; during PRRT, reduced to ~–0.3%/mo (stabilization).
[35]	Observational retrospective study	48 GEP-NET (G1-G3)	High baseline TGR is associated with shorter time to treatment, worse OS, and increased Ki-67 during follow-up.	Cut-off: >11.7%/month defined “high TGR”.
[15]	Multicenter retrospective study	127 NET (G1-G2)	TGR validated as early biomarker reflecting treatment-induced changes and predicting PFS/response.	No fixed threshold; dynamic changes in TGR are used for prediction.
[36]	Multicenter retrospective study	22 GEP-NET (G1-G2)	TGR at 3 months (TGR_3_m) is predictive of PFS and identifies high-risk patients needing closer follow-up.	TGR_3_m increase associated with poor outcomes; no single universal cut-off, but rising TGR = adverse.
[37]	Retrospective cohort study	198 GEP-NET	Approach combining tumor growth and regression parameters captured worse PFS.	A reduction of 26% in TGR was related to significantly higher tumor volume doubling time.
[38]	Multicenter retrospective cohort study	288 PanNET	SGR, mathematically related to TGR, was independently associated with OS. Patients with higher SGR had significantly worse outcomes, supporting its role as a prognostic biomarker.	High SGR (positive growth) correlated with poorer OS; exact cut-offs varied, but SGR > 0 defined progressive disease.

NET: neuroendocrine tumors; GEP-NET: gastroenteropancreatic neuroendocrine tumors; PanNET: pancreatic neuroendocrine tumor; SGR: specific growth rate; TGR: tumor growth rate.

## Data Availability

No new data were created or analyzed in this study. Data sharing is not applicable to this article.

## Abbreviations

The following abbreviations are used in this manuscript:

**Acronym**	**Definition**
AUC	Area under the curve
CR	Complete Response
CT	Computed Tomography
cTGR	Cylindrical Tumor Growth Rate
DT	Doubling Time
L-NEN	Lung-Neuroendocrine neoplasm
MEN1	Multiple Endocrine Neoplasia type 1
MRI	Magnetic Resonance Imaging
mTOR	Mammalian target of rapamycin
NEN	Neuroendocrine neoplasm
ORR	Overall Response Rate
panNET	Pancreatic NET
PD	Progression Disease
PR	Partial Response
PRRT	Peptide receptor radionuclide therapy
RECIST 1.1	Response Evaluation Criteria in Solid Tumors version 1.1
RLT	Radioligand therapy
SD	Stable Disease
SGR	Specific Growth Rate
SSA	Somatostatin Analogs
TGR	Tumor Growth Rate
TKI	Tyrosine Kinase Inhibitor
WHO	World Health Organization
